# Psychedelic Research and the Need for Transparency: Polishing Alice’s Looking Glass

**DOI:** 10.3389/fpsyg.2020.01681

**Published:** 2020-07-10

**Authors:** Rotem Petranker, Thomas Anderson, Norman Farb

**Affiliations:** ^1^Clinical Psychology, York University, Toronto, ON, Canada; ^2^Psychedelic Studies Research Program, University of Toronto Mississauga, Mississauga, ON, Canada; ^3^Department of Psychology, University of Toronto, Toronto, ON, Canada

**Keywords:** psychedelics, Open Science, research practices and methods, checklist, psilocybin, LSD, replicability, transparency

## Abstract

Psychedelics have a checkered past, alternately venerated as sacred medicines and vilified as narcotics with no medicinal or research value. After decades of international prohibition, a growing dissatisfaction with conventional mental health care and the pioneering work of the Multidisciplinary Association for Psychedelic Science (MAPS) and others has sparked a new wave of psychedelic research. Positive media coverage and new entrepreneurial interest in this potentially lucrative market, along with their attendant conflicts of interest, have accelerated the hype. Given psychedelics’ complex history, it is especially important to proceed with care, holding ourselves to a higher scientific rigor and standard of transparency. Universities and researchers face conflicting interests and perverse incentives, but we can avoid missteps by expecting rigorous and transparent methods in the growing science of psychedelics. This paper provides a pragmatic research checklist and discusses the importance of using the modern research and transparency standards of Open Science using preregistration, open materials and data, reporting constraints on generality, and encouraging replication. We discuss specific steps researchers should take to avoid another replication crisis like those devastating psychology, medicine, and other fields. We end with a discussion of researcher intention and the value of actively deciding to abide by higher scientific standards. We can build a rigorous, transparent, replicable psychedelic science by using Open Science to understand psychedelics’ potential as they re-enter science and society.

## Brief History

After decades of dormancy, a new generation of psychedelic science is emerging. During the 1950s and 1960s, the popularity of psychedelics was soaring with more than 40,000 people being administered LSD between 1950 and 1965 (Kabil, 2016). Promising therapeutic effects of psychedelics were described in varied conditions, such as end-of-life anxiety (Kast, 1966), depression, and alcohol use (Pahnke et al., 1970), as well as reports describing psychedelics used to enhance creative problem solving in complex decisions (Harman et al., 1966). Literary figure Aldous Huxley introduced a naïve public to the phenomenology of psychedelic experiences in “The Doors of Perception” (1952). Much of this pioneering research was sponsored by the National Institute of Mental Health, attesting to the institutional support that psychedelics enjoyed.

Despite promising results, psychedelics soon became illegal. A combination of poor knowledge translation efforts, rising conservatism in the face of international conflicts, and misuse of psychedelic substances by high-profile researchers led to their global prohibition in 1971 (Dyck, 2008; UNODC, 2013; Pollan, 2018).

**FIGURE 1:**
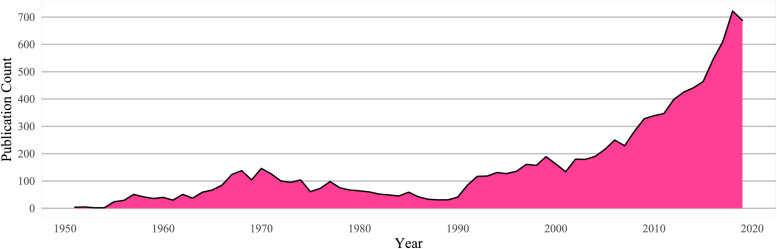
Web of science psychedelic publication count by year.

Prohibition resulted in suppressed publication rates for psychedelic research for decades following initial growth in the 1950–1960s: a web of science reviews searching for “LSD,” “PSILOCYBIN” (the active ingredient in “magic” mushrooms), “PSYCHEDELICS,” or “HALLUCINOGENS” shows the effects of decades of prohibition on a nascent field (Figure 1).

Despite ongoing prohibition, the twenty-first century has seen a resurgence in psychedelic research, fueled by promising early work at Johns Hopkins University and Imperial College London (Griffiths et al., 2006; see Goldberg et al., 2020 for a review). This return is a testament to decades of lobbying by researchers and organizations dedicated to bringing psychedelics back under the umbrella of legally sanctioned research, such as the Multidisciplinary Association for Psychedelic Studies (MAPS), the Beckley Foundation, and the Heffter Institute. High quality peer-reviewed clinical trials have demonstrated the potential for larger-dose psychedelics in contexts of treatment resistant depression, anxiety, and substance use disorders (dos Santos et al., 2016). A second branch of research explores psychedelics’ potential for well-being enhancement in non-clinical samples, including recent research on the growing practice of taking psychedelics in very small doses, called “microdosing” (Prochazkova et al., 2018; Anderson et al., 2019; Polito and Stevenson, 2019; Rosenbaum et al., 2020).

The resurgence of psychedelic research coupled with profits from the cannabis legalization has led to converging clinical, philanthropic, financial, and private industry interest in psychedelics. Additionally, recent mainstream media coverage of psychedelic research has been positive, which has increased public and political interest in the topic. Scientists may soon be called upon to provide evidence pertinent to policy decisions regarding compassionate-use exceptions, decriminalization options, and legalization frameworks. This spotlight behooves psychedelic researchers to focus on rigor and transparency in evaluating drug efficacy to avoid sensationalism over tentative findings (c.f. Godlee et al., 2011), on the unsubstantiated link between vaccines and developmental disorders). With psychedelics, researchers should exercise an even-handed caution against extrapolating too far from the research literature: this is a field characterized by both pro- and anti-drug agendas. Mainstream narratives are fragile: current excitement favoring psychedelics’ potential could swing swiftly back toward prohibition rhetoric following the emergence of clinically adverse events. Peer-reviewed research provides a more stable foundation for policy recommendations, but only when the research is rigorous and transparent. Based on our early experiences in this field, we propose a research checklist for psychedelic scientists to optimize the rigor and transparency of their research (see Appendix) and we provide personal considerations for interacting with common stakeholders in the broader social and research process.

## Research Checklist

The need for these recommendations is supported by the replication crisis in psychology, biology, medicine, and other fields, in which researchers are pressured to produce positive, novel research findings in a “publish or perish” academic environment (Frankenhuis and Nettle, 2018; Shrout and Rodgers, 2018). Describing all the ways in which long-term research goals can be undermined by short-term Questionable Research Practices (QRPs) is outside the scope of this paper; we recommend the work of others (Ioannidis, 2005, 2012, 2014, 2016; Munafò et al., 2017) as a primer on the greatest concerns.

While the following checklist can be applicable to experimental research including fields such as pharmacology, biology, and medical science, our intention here is to establish standards that will improve research quality in psychedelic science in particular. While not exhaustive, it provides Open Science principles that can then be implemented as necessary to reflect best practices in different fields. Johnson et al. (2008) provide researchers with valuable guidelines focused on participant safety given the special nature of these substances; the present article compliments this work by providing researchers with a methodologically focused checklist highlighting the important role their research will play in policy and underscoring the public attention placed upon psychedelic science. We hope the following checklist will establish reasonable standards for psychedelic researchers to consider when designing studies and implementing research paradigms.

There are several expected benefits to following a standard psychedelic research checklist: it will create more robust, transparent science with clear standards for evidentiary claims; it will protect scientists as skeptical policy makers and institutional gatekeepers question their research; it will help clearly communicate the limitations of research to the already overzealous public, private, and media stakeholders, all while maintaining an excitement for the potential of psychedelics. Given the complexity of research, this checklist may not be universally applicable, but researchers should endeavor to follow this checklist whenever possible. By following this checklist, we can avoid a replication crisis in psychedelic science and ensure our time, effort, and funding are well spent.

The following research checklist highlights four key areas to enhance rigor and transparency: Pre-registration, Open Materials and Open Data, Constraints on Generality, and Replication. We begin with the most crucial step for establishing trustworthy research: pre-registration. The Appendix contains a full checklist of actionable items.

### Pre-registration

Psychedelic science may be particularly vulnerable to QRPs due to the presence of both ideological and financial conflicts of interest. By ideological conflicts, we mean research stakeholders who are motivated by faith in the utility of psychedelics and are therefore resistant to disconfirming evidence. By financial conflicts, we mean research sponsors whose livelihoods are benefited by the commercial viability of psychedelics, which demands that the drugs be effective and safe. Both of these approaches may run counter to the scientific method and should therefore be tempered with a commitment to transparently reporting results. Scientists should set out with a curious approach and the intention of studying the effects of these substances to discover what is true about them, for good and ill. We should be honest about effects, be they positive, negative, or null. To prevent undue commitment to career, ideological, financial, or other perverse incentives, we encourage psychedelic scientists to pre-register all future research.

Pre-registration is different from registration on websites such as clinicaltrials.gov in that it is a much more comprehensive process which includes a description of all hypotheses, planned analyses, and sample size. That means planning and uploading a complete, date-stamped experimental design and analysis plan before human observation of data. Ideally, pre-registrations should be completed before data-collection begins, though this is not always practical. At a minimum, scientists should pre-register before data is observed and before running analysis; this ensures confirmatory analyses are distinguished from exploratory analyses. We recommend pre-registering methods and analysis plans in as much detail as possible by using the fillable forms on free, reputable repositories, such as the Open Science Framework^1^.

Pre-registration commits the researcher to formally stating hypotheses, procedures, and collected variables, establishing an analytic/statistical plan, rules for data exclusion, and sample-size stopping rules. Unlike the common misconception, pre-registering does not preclude exploratory or explanatory *post-hoc* analysis. Investigators can probe data following pre-registered analyses, so long as these efforts are explicitly labeled as exploratory rather than confirmatory. It is also acceptable to deviate from a pre-registration, if deviations are explicit and transparent. Rather than limit analysis, admitting that some analyses are exploratory liberates the researcher from overly conservative error correction, increasing study power for making statistical inference around *a priori* hypotheses. By explicitly labeling exploratory research as such, research can earmark results for future validation rather than fueling premature conclusions.

Pre-registration may sound like a daunting process. There is a learning curve, but pre-registering makes workflows more efficient in the long run. Pre-registering requires researchers to solve problems in the planning phase rather than only discovering issues after data has been collected. Researchers can conduct *a priori* power analyses and consider whether variables being collected given practical constraints on sample size, and ensure that their design and analysis can answer their research questions adequately. Pre-registration also protects the researcher and the broader scientific community from reliance on improperly liberal thresholds for statistical significance and from hypothesizing after the results are known (HARKing; Kerr, 1998), which directly contribute to contemporary replicability issues.

### Open Materials and Open Data

The second checklist item asks researchers to provide study materials and collected data so that reviewers and other scientists can evaluate, reproduce, replicate, and explore the methods and data used to determine research findings.

*Open materials* is the process by which all questionnaires, stimuli, and tasks used in the methods section of a paper are made available publicly, without having to contact the researcher. This practice is particularly important for the study of psychedelics where many measures have not been separately validated, but instead are composed *ad hoc* to address the needs of a study (e.g., Psychological Insight Questionnaire, Carbonaro et al., 2018). By sharing even preliminary, draft questionnaires and tasks we can expedite new measure validation. Moreover, anyone interested in replicating existing experimental designs could easily use the same measures so that new research can be directly compared to the extant literature. As replication is a cornerstone of the scientific method, researchers should provide all the materials needed to run an identical study. Some materials may be copyrighted in which case they may not be readily shareable, but researchers should provide as much as they can and try to rely on open measures. Open materials is instrumental to the replicability and interpretability of psychedelic research.

*Open data* asks researchers to share a complete, de-identified dataset of all data collected as part of their study. Researchers should provide the data that was used in the analysis for a given paper in a form such that another scientist could re-run their analyses and reproduce the findings in the paper. Scientists often have reservations about sharing data, some reasonable and others unfounded. For example, when handled properly, sharing de-identified data is generally acceptable to Research Ethics Boards so long as participants have consented to having their de-identified data made available. Researchers sometimes worry that someone else could access their data and publish before them; we do not suggest that researchers make their data available before publication. Instead, researchers can publish their data alongside their first publication. Some researchers may have especially large data-sets designed to produce many papers and may choose to publish their data piece-meal. The researcher should take care, however, to limit how long they keep data private. We recommend that data be held privately for no longer than 2 years before sharing. This gives researchers ample time to write and publish their research while also preventing data from ending up in the file drawer. This is even more important when a researcher provides results counter to hypotheses or convention, including null results, as these findings are less likely to be easily publishable and thus the researcher may feel less incentivized to work on publishing them. Open data ensures all data see the light of day.

Openly sharing materials and data with the scientific community benefits us in many ways. Primary analyses can be rerun, verifying that no errors were made. Novel exploratory analyses can be attempted without barriers to access and new hypotheses can emerge from exploring existing open datasets, encouraging collaboration and follow-up investigations. Open materials and data is even more important for expensive neuroimaging where data is scarce and researcher degrees of freedom are high, resulting in especially limited replicability (Button et al., 2013; Szucs and Ioannidis, 2017).

### Replication

Replication is particularly important for the nascent field of psychedelics research as the resurgence in research could come at the expense of building a strong foundation. Replication ensures that foundational scientific research is solid and provides an opportunity to work together as a community of researchers. Replication only requires that we repeat the same experimental design others have used to test whether results are similar across samples and labs. Current psychedelic research provides fertile ground for replication: most researchers are using similar interventions (psilocybin/LSD) to treat a small number of disorders (depression, anxiety, or substance use). For example, a researcher interested in treating depression with psilocybin could include a questionnaire, measure, or dose-condition used by other researchers who had previously studied the effects of psilocybin on depression so that results can be compared.

Replication efforts are perhaps the only way the scientific community can effectively test for Type-I error, i.e., whether promising findings were obtained by chance despite pre-registration and a strong commitment to scientific rigor. If results fail to replicate, we should regard them as less reliable, and consider looking for smaller effect sizes or using higher-power designs in the future. Failures to replicate are not necessarily dichotomous; instead, researchers should evaluate whether effect size estimates ought to be updated rather than wholly accepted or rejected, leading to more specific research designs and theories. Using the same measures or conditions also makes meta-analyses much easier to compute. We recommend that every new study aims to replicate at least some of the work on which it is based.

### Constraints on Generality

Simons et al. (2017) recommend that all empirical papers include a “constraints on generality” (COG) section, which serves as a structured replacement for the limitations section usually included in a paper. This elegant solution serves many purposes, including ensuring important limitations are not overlooked and explicitly incorporating incremental theory-testing and development into empirical papers. In brief, a COG section should explicitly identify qualities of participants, materials, procedures, and context that the researchers consider necessary and/or sufficient for observing the reported effects; they should also explicitly identify qualities thought to be irrelevant, i.e., over which the results are generalizable. Such explicit description allows the researcher to describe what qualities are needed for a future study to be considered a direct replication or a conceptual replication. COG sections should explicitly consider boundary conditions as regards the study itself and the context surrounding the study (e.g., use of elderly participants) and should include “known unknowns,” such as moderators of potential importance that were not manipulated (e.g., whether participants were psychedelics-naïve).

For a practical guide on what specific questions should be considered when writing a COG section, we defer to Simons et al. (2017). An example as pertains to psychedelics is offered as Supplemental Materials. Overall, the structure of a COG statement reminds researchers to perform a due diligence to identify qualities of the messy reality of research that may play a role in limiting what we can learn from a study. Reporting limitations in a well-designed study does not undermine the findings therein; limitations lend context for other researchers to take research for what it is: a real report on a small slice of reality designed to inform how we view a broader slice of reality. We will be greatly served by more clearly delineating what we have learned from what we suspect and what we know matters from what we think but don’t know.

Yarkoni (2019) also highlights the need for thoughtfulness and restraint, especially as relates to statistical and theoretical inferences drawn from experiments of necessarily limited scope. Psychedelics show great promise, but we must remember that most contemporary lab studies have small sample sizes and that larger survey studies, including our own, are necessarily limited by lack of experimental control. Such studies, even with their exciting statistically significant results, do not “prove” the benefits attributed to psychedelics so we should hedge our expectations and pronouncements. Replications are needed, as are iterations on and manipulations of various experimental qualities thought to be important (or irrelevant) to any intervention so we may determine what matters (Yarkoni, 2019). Reviewers are also encouraged to embrace descriptive data reporting for novel areas where statistical inferences may not yet be appropriate, favoring effect-size estimates with confidence-intervals over *p*-values (Cumming, 2014) and COG statements should endeavor to make explicitly falsifiable predictions whenever possible. By embracing falsifiability through replications and extensions that could imply a need to modify our theories, psychedelic science would be set for fruitful, collaborative theory development and refinement.

## Commitment to Scientific Rigor and Transparency

Few people worry about participant safety and legal liability in a computer-led task measuring memory, but safety questions are present at every level of formal oversight and regulatory approval for the psychedelic scientist. Conversely, few researchers of traditional topics are bombarded by lay-people offering to provide illicit study materials or unregulated funds, seeking to bypass bureaucratic constraints and institutional oversight. High-quality psychedelic research must protect itself from both overzealous institutional safety concerns and from private interests that wish to skirt regulation in favor of fast results or market advantage. Since launching the Psychedelic Studies Research Program at the University of Toronto about a year ago, we have been flooded with emails, phone calls, offers of money with ambiguity around the strings attached, and offers of illegally cultivated or synthesized substances to help speed our research along.

Our experiences have taught us that a psychedelic scientist must decide what principles they will uphold. Lucrative offers are readily available for the researcher who is willing to contribute to the hype surrounding psychedelic science, typically at the expense of certain publishing rights and intellectual freedoms. Such offers may include verbal promises to commit to scientific rigor that disappear when the legal agreement is drafted. Psychedelic researchers must ask themselves whether they have responsibilities to their communities. Is it the job of a researcher to safeguard against exaggerated claims? Should a researcher call out bad science when they see it? How will the researcher respond if their data goes against the desires of their financial stakeholders?

We are committed to the principles of Open Science and we believe that the principles of rigor and transparency will provide the most fruitful long-term prosperity of psychedelic science, especially as they are embraced by more researchers. Working within legal and institutional frameworks is absolutely essential to avoid the mistakes of the past. By prioritizing transparent and accurate reporting to the broader scientific, psychedelic, and public communities, we will make psychedelic science a credible, durable science fit to inform legal and medical policies in the years to come.

## Conclusion

Research done by psychedelic pioneers in the 1950s and 1960s has been instrumental to current work in the field. While we cannot control the political tides, we can try to make sure that politicians make informed decisions based on good research. We propose that pre-registration, open materials and data, constraints on generality, and replication are best practices for any scientific endeavor and particularly for psychedelics research. We recommend working with a university or other recognized institution when studying scheduled substances, obeying the law, and hiring legal counsel to review potential agreements with industry partners. This is an exciting time to study psychedelics, but getting the science right should be our top priority.

## Data Availability Statement

Publicly available datasets were analyzed in this study. This data can be found here: Web of science citation counts, sorted by year.

## Author Contributions

RP, TA, and NF contributed equally to the conceptualization, drafting, revisions, and final approval of this perspectives piece. RP led writing of the first draft. All authors contributed to the article and approved the submitted version.

## Conflict of Interest

The authors declare that the research was conducted in the absence of any commercial or financial relationships that could be construed as a potential conflict of interest.

## Footnotes

1 http://osf.io
