# Supplementary material for: Psychedelic Research and the Need for Transparency: Polishing Alice’s Looking Glass
Source: Front Psychol. 2020 Jul 10;11:1681. doi: 10.3389/fpsyg.2020.01681 (PMC7367180; doi:10.3389/fpsyg.2020.01681)
Supplement: Supplementary file 1 [file Data_Sheet_1.docx]

# Appendix – Research Rigour and Transparency Checklist

**Preregistration:**

***Before human observation of data:***

- Register hypotheses, including direction of effect; estimate effect-size if possible
- Register all measured variables, including scales and scoring methods
- Register intended sample size and stopping rules, whether due to power analysis or feasibility
- Register statistical analysis plan, including type of modelling and confirmatory statistical tests; include as much detail about modelling decisions as feasible
- Register data exclusion rules and rules for handling missing data (e.g. pairwise deletion, imputation)
- Register planned follow-up or exploration if known in advance

***For publication and presentation:***

- Report all confirmatory analyses as registered
- Report all deviations from pre-registration
- Report exploratory analyses as exploratory

**Open materials and data:**

- Include data-sharing in participant consent form and ethics application
- Use non-copyrighted materials if possible
- Share custom materials and new scales on an accessible platform with instructions
- Share other materials on an accessible platform unless copyright prevents sharing; if copyrighted, specify where materials are available
- Note any modifications to existing materials
- Create de-identified version of data for sharing, i.e. remove identifying information
- Include descriptive text document to decode all variables
- Share data on an accessible platform, e.g. OSF

**Constraints on generality**

- Report sample characteristics
- Report which sample characteristics may moderate results
- Report which sample characteristics should not moderate results
- Report theory characteristics
- Report edge cases for theory to indicate blind-spots
- Report primary predictions of theory to indicate falsifiable hypotheses
- Report what evidence would require the theory be revised

**Replication**

- Register which finding(s) from which previous research will be subject to the replication attempt(s)
- Register expected direction and effect size
- Register similarities and differences from original study
- Report and compare original effect-size and replication-attempt effect-size
- Interpret confidence in effect given the additional data
